# Provision of Care to Preterm Infants at Resource Limited Health Facilities of Mopani District, South Africa

**DOI:** 10.5334/aogh.2555

**Published:** 2020-02-03

**Authors:** Thendo Mahwasane, Maria S. Maputle, Khathutshelo G. Simane-Netshisaulu, Thivhulawi Malwela

**Affiliations:** 1 Department of Advanced Nursing Science, University of Venda, ZA

## Abstract

**Background:**

Inadequate neonatal facilities in rural areas is one of the challenges affecting the management of preterm infants. In low income countries with limited resources, over 90% of preterm babies die within few days of life.

**Purpose:**

The purpose of this study was to describe the challenges encountered by midwives when providing care to preterm infants at resource limited health facilities in Limpopo Province, South Africa.

**Methods:**

Qualitative research approach, using exploratory and descriptive design was used. Non-probability purposive sampling was used to select twenty three midwives who had an experience of two or more years in maternity. Data was collected using unstructured individual interviews, which were voice recorded and transcribed and data analysed qualitatively through the open-coding method.

**Findings:**

Revealed one theme, preterm condition and expected care; with sub-themes namely; perceived causes of preterm complications and deaths, preterm babies experience several difficulties which need specialised care, the need for constant individualised care and monitoring of preterm infants by midwives, functional relevant equipment needed for care of preterm infants, a need for constant training for midwives regarding care of preterm infants, and importance for a proper structure to house preterm infants which will lead to quality care provision.

**Conclusion:**

Preterm babies need simple essential care such as warmth, feeding support, safe oxygen use and prevention of infection. Lack of adequate resources and limited skills from midwives could contribute to morbidity and mortality. Health facility managers need to create opportunities for basic and advanced preterm care to equip the skills of midwives by sending them to special trainings such as Limpopo Initiative Neonatal Care (LINC), Helping Baby Breath (HHB) and Neonatal Intensive Care Unit (NICU). Operational managers should be involved in the identification, procurement and supply of required equipment. Continuous health education should be provided on the mothers about kangaroo mother care (KMC) and measures to prevent infections in the neonatal unit.

## Introduction

The Perinatal Problem Identification Programme (PPIP) produced reports which highlighted the preventable deaths in South Africa. Every year about 23,000 new-born babies die in South Africa, with an additional estimated 20,000 stillbirths [1]. Forty-five percent of these babies die from preterm-related complications [1]. In the 2006–2007 Perinatal Care Report of South Africa, preterm birth (PTB) was found to be responsible for 46% of all neonatal deaths followed by 29.8% of asphyxia [2]. This observation was supported by the Department of Health 2012–2013 Saving Babies Report, Pattinson and Rhoda [3]. Pattinson alluded to the lack of adequate neonatal facilities in rural areas as one of the challenges in the management of preterm infants; wherein, women arrive at health care facilities being in advanced labour and the suppression of labour is no longer an option [4]. Delivery of the preterm baby becomes imminent. The prevention of preterm labour is one of the greatest challenges for midwives [5]. Patel et al. recommended that child survival strategies should direct resources toward the leading causes of child mortality which are pneumonia and preterm birth complications [6]. According to a study conducted in Limpopo Province in 2003 by UNICEF to ascertain the status and availability of new-born care services and infrastructure, it was found that none of the health facilities had level 2 new-born care units, few nursing staff were trained in new-born care and many health facilities had inadequate equipment to provide standard quality of care to new-borns [7]. These factors lead to midwives’ challenges as reflected in the Saving Babies 2012–2013 report [8]. There were delays in seeking medical attention which accounted for most immaturity deaths (10.5%), never initiated ANC (8.8%), late booking (3.1%), inappropriate response to poor foetal movements (2%), inadequate neonatal care management plan (2%), and not giving antenatal steroids (1.2%) [3]. Midwives could play a major role in the prevention of these factors. Pattinson found that lack of adequate neonatal facilities were the most common administrative problem that caused deaths amongst preterm babies [9]. In low income countries with limited resources, over 90% of preterm babies die within few days of life [10]. Preterm Birth (PTB) is the first leading cause of child death after pneumonia [10]. This was supported by WHO, that 15 million babies are born too soon every year and over 1 million children die each year due to complications of PTB [10]. PTB is the significant cause of short and long-term morbidity which increases the demand for neonatal intensive care services. A large number of surviving preterm babies tend to end up with deficits such as blindness, neurological impairment and chronic respiratory distress [11].

Preterm babies lose heat very rapidly after birth [12]. These babies need simple essential care such as warmth, feeding support, safe oxygen use and prevention of infection which can be achieved by use of chlorhexidine and avoiding sharing of incubators for neonates [11]. However, in a limited resource setting there are limited incubators. Premature babies are vulnerable to temperature instability, feeding difficulties, low blood sugar, infections and breathing complications [11]. About 10% are born in low income settings with limited physical and human resources barely [12]. Midwives caring for preterm infants receiving mechanical ventilation, faces many challenges. Important aspects of care to be carried out by midwives include thermoregulation, optimal positioning, airway clearance, stable haemodynamic status, and adequate nutrition for growth and development [13]. Midwives face challenges of staff shortage when working at the state hospitals [14]. This was evidenced by findings of a study entitled “Occupational challenges faced by nursing personnel at a state hospital in Cape Town, South Africa” by Brophy who found that midwives face challenges of providing quality maternal and neonatal care due to poor working conditions, feeling of insecurity and frustration at work [14]. Thommesen revealed that most midwives in Ethiopia started in midwifery without a passion and they do not show urgency in emergency situations [15]. Brophy reflected on a challenge of lack of resources [15]. It was found that if a hospitals’ resources were not well managed, the service delivery to patients would be negatively affected. The use of open multi-patient rooms posed a challenge in a manner that it increases the costs of care for preterm babies who are kept in NICU. The use of private and semi-private rooms for neonates allows for improved care resulting in reduced hospital stay and lower costs for NICU. Prolonged length of hospital stay was one factor that attributed to this increased NICU costs. Complications that increase the length of stay adversely affected costs. However, therapies that reduce the intensity of illness decrease the costs of care. Reduction of length of hospital stay can be achieved by minimising hospital-acquired infections which usually occurs during close contact between patients, preterm babies in this case and accidentally using the same equipment in infants.

Shortage of nurses in neonatal care is also a challenge [16]. Perinatal deaths from spontaneous preterm labour is sometimes caused by lack of adequate neonatal facilities in rural areas wherein the woman arrives to the institution in advanced labour and the baby is delivered shortly thereafter, the opportunity for interventions by suppressing labour and giving corticosteroids is therefore low [17,18]. This study was conducted in the Mopani District which is one of the five districts of the Limpopo Province. The statistics of January–June 2016 showed that the perinatal mortality rate per 1,000 in Mopani hospitals were as follows: 59.7% in Maphutha Malatji; 37% in Letaba; 36.1% in Sekororo; 35.6% in CN Phathudi, 44.1% in Kgapane; 34.8% in Nkhensani and 30.9% in Van Velden Hospital [17]. Hence, this research focused on describing the challenges encountered by midwives when providing care to preterm infants.

## Methodology

### Study setting

The study was conducted at three purposely selected district hospitals of Mopani District in Limpopo Province.

### Design

The qualitative research, exploratory, descriptive design were used in order to gain an insight and more understanding regarding challenges encountered by midwives when providing care to preterm infants.

### Population and sampling

The population comprised of twenty-three midwives who have worked in maternity wards for not less than two years and who gave consent to participate in the study. Non-probability purpose sampling was used to select participants at three hospitals (Letaba (A), Kgapane (B) and Maphutha L. Malatji (C) with limited resources of Mopani district.

### Data collection

Data was collected by the researcher at respective hospitals during March to September 2017 in the duty rooms during lunch time. Unstructured face-to-face interviews was used. The central question directed interviews was: *“What challenges have you encountered when providing care to preterm babies in this unit?”* Interviews were conducted in English for 45–60 minutes, in the duty room during the resting period of participants. A voice recorder was used to capture information and a probing method was used to deepen the responses to questions and to increase the richness of the data [19]. Interviews were conducted until data saturation was reached.

### Data analysis

Data was analysed using the six steps as described by Creswell [20]. The method included the following steps: The researcher listened to the recorded interviews, transcribed and arranged with the written data. The researcher then read all the data to get sense and meaning. After reading, coding was done using the eight steps provided by Tesch’s (1990) in Cresswell [20]. The researcher wrote information under the themes and sub-themes supported by the participants’ quotes.

### Trustworthiness

The researcher ensured the scope, depth quality of data by employing the four criteria of trustworthiness as outlined in Lincoln and Guba [21]. Trustworthiness was ensured through using criteria of credibility, transferability, dependability and confirmability. Credibility was ensured by prolonged engagement to establish rapport and build trust. During the interviews, the researcher spent time with the participants listening and observing them as they were interviewed. The participants were interviewed to the point at which there was data saturation. Member checking was also done formally after data had been fully analysed. Thus, the preliminary findings of the research were discussed with the participants to validate the results. Referential adequacy was achieved by taking notes to record findings that provided a suitable record, and the use of a voice recorder. Dependability was ensured by assigning the independent coder to deal with the raw data including the field notes to come up with themes independently and an agreement was reached with the researcher for final themes. Transferability was ensured by thick descriptions of research methodology. Confirmability was ensured by having an assistant to transcribe the same data and then checked if the data reconciled. The recorded interviews were transcribed word by word and the nonverbal cues (for example, silence/sigh, frowns, and lean back) were included in brackets of the transcripts to ensure authenticity.

## Ethical considerations

The ethical clearance SHS/17/PDC/04/1403 was approved by Ethics Committee of University of Venda. Permission (Ref: 4/2/2) to access the selected hospitals was obtained from Limpopo Department of Health. Informed consent to participate in the study was obtained from each participants. An informed consent form was completed by all participants, which evidenced that they participated in the study willingly without being coerced. Emphasis was on confidentiality and anonymity which indicate that only the researcher and the research team during the research process should be aware of the identity of the participants.

## Findings

Twenty-three midwives who had an experience of at least two years of working in maternity ward were interviewed from three selected hospitals with limited resources. Distribution of participants were as follows; 8 from hospital A, 8 from hospital B and 7 from hospital C. The results revealed one theme with sub-themes as presented in Table 1.

**Table 1:** Theme and sub-themes as challenges of providing care to preterm babies.

Theme	Sub-themes

1. Preterm condition and expected care	1.1. Perceived causes of preterm complications and deaths 1.2. Preterm babies experience several difficulties which need specialised care 1.3. The need for constant individualised care and monitoring of preterm infants by midwives 1.4. Functional relevant equipment needed for care of preterm infants 1.5. A need for constant training for midwives regarding care of preterm infants 1.6. Importance for a proper structure to house preterm infants which will lead to quality care provision

### Demographic data of participants

Females constituted 95.7% of all the participants and males 4.3%. In terms of ethnic groups, 47.8% were Pedi, 39.1% Tsongas, 8.7% Swati and 4.3% Venda. With regard to occupation, 4.3% were midwives who were operational managers and 95.7% general midwives. Of all the participants, 65.2% had midwifery experience of 5 years and above and the other 34.8% less than 5 years, and none of the midwives had been trained on NICU or Paediatric Nursing Sciences. Further, no participant was found to have attended LINC and HBB formally, but attended it as in-service training.

The emerged theme and sub-themes are then discussed in line with the results of the study.

## Theme 1: Preterm condition and expected care

Preterm infants experienced multiple complications and midwives encountered challenges when managing those conditions due to limited resources. This was evidenced from the interviews.

*“There is only one ventilator machine, four duopaps and two nasal Continuous Positive Airway Pressure (nCPAP) machines; and the Neonatal Intensive Care Unit (NICU) is having eight beds and only very ill babies are admitted in that unit. Most of the babies being admitted are preterm babies with severe respiratory distress syndrome (RDS) and they will need to be assisted by these machines for them to be relieved from RDS. When a preterm baby is not coping on duopap and nCPAP machines, they should be initiated on a ventilator machine, but it is a challenge because the machine is only one. The unavailability of ventilator machine poses a challenge because care for another baby will be compromised”* (Participant G from hospital A).

Sub-themes which are presented in Table 1, emerged from this theme.

### Sub-theme 1.1: The causes of preterm complications and deaths

The complications of preterm birth arise from immature organ systems that are not yet prepared to support life in the extra-uterine environment. Respiratory Distress Syndrome (RDS) related to prematurity accounts for 15% morbidity in infants born at 34 weeks and 3.2% in those born at 36 weeks [22]. The following citation made by participant number 1 from hospital A was in agreement:

“…Preterm babies suffer from RDS which is a common condition in these babies, it is due to immaturity of the lungs and it is the most common cause of death in premature babies, more especially in those who were born at less than 1 kg. Conditions that are related to prematurity and may cause inadequate weight gain are neonatal jaundice, infection and feeding intolerance. They take long to gain weight due to the conditions that they may be diagnosed with during their stay in the hospital.”

There was higher incidence of perinatal mortality amongst preterm babies.

### Sub-theme 1.2: Preterm infants experience several difficulties which need specialized care

The immaturities of the systems in preterm babies lead to many complications that affected multiple systems and which need specialised care. Participants indicated that each clinical problem may lead to more complicated conditions, if not managed correctly and in time. This was supported by the following citations from participants;

*“Preterm babies are very prone to infections. Although to some, antibiotics are given to prevent infections. We teach the mothers and other staff members the practice of antiseptic techniques for hand washing with soap and clean water … rub hands with alcohol and chlorhexidine solution (D-Germ®) before and after handling the baby.”* (Participant 7 from hospital B).

*“… because of their systems which are not well developed, premature babies suffer from hypothermia which can result into more serious conditions like hypoglycaemia and respiratory distress. The hospital had limited resources like to practice kangaroo mother care, or incubators to keep babies.”* (Participant 5 from hospital B).

*“Due to immature liver, preterm babies are most likely to be diagnosed with neonatal jaundice (NNJ) within their first days of life. Babies with NNJ are nursed under phototherapy lamps and should be collected blood daily to see if the condition is healing or not. We have only one functional phototherapy lamp. If we can have more than one babies, it was difficult to provide care. The veins of these babies are very small and they sometimes disappear and become invisible which makes it hard to collect blood, remember we are not trained as neonatal nurses.”* (Participant 1 from hospital B).

These babies need simple essential care such as warmth, feeding support, safe oxygen use and prevention of infection which can be achieved by use of chlorhexidine and avoiding sharing of incubators for neonates [9]. However, these hospitals had only the maximum of two functional incubators and more infants who would need to be kept in the incubator. Kangaroo care was promoted in all the hospitals. In one hospital, the special dress was designed to facilitate kangaroo care.

### Sub-theme 1.3: The need for constant individualized care and monitoring of preterm babies by midwives

Preterm babies need individualised, close and constant monitoring as they were always changing condition and care should not be routine. It was very challenging for midwives to care for preterm babies as they did not always respond the same way to the same treatment. This was supported by the following quotes from participants;

*“Preterm babies need to be monitored constantly. As midwives, (but not trained as neonatal nurses) we need to be skilled and be alert about the conditions associated with prematurity. This necessitated, the hospital to have sufficient supplies and equipments, however, the shortages affected this goal.”* (Participant 6 from hospital B).

*“We admit lots of babies and they need individualized care as they don’t respond the same way in treatment. Each baby will need to be treated according to their current condition, age and weight, but we are not managing due to lack of resources.”* (Participant 7 from hospital C).

If midwives are competent, confident then individualised care could assist in the provision of care and health education to the mother, diagnose early, and for appropriate management of preterm infants.

### Sub-theme 1.4: Functional relevant equipments needed for care of preterm babies

Equipments such as incubators, pulse oxymeters, ambubags, oxygen apparatus, should be available and in good working order for midwives to execute quality care to the preterm babies. Without the required equipments, nursing care was compromised. What was more challenging to the midwives who were directly providing care to these preterm babies were limited or non-functional equipments. The following citations were expressed by the midwives:

*“…each bed should have a pulse oxymeter to continuously monitor the heart rate and oxygen saturation, but we are having only two and one of them does not function well”* (Participant 2 from hospital A).

*“These babies have a problem of hypothermia, they cannot maintain their own normal body temperature because of lack of brown fats in their body, and they should be nursed inside warm incubators, however, at our hospital only 2 incubators are working. Some preterm babies need to be kept incubator before they are placed to KMC.”* (Participant 3 from hospital C).

Some midwives indicated situations where equipments for resuscitating were available but since the preterm babies were small, they needed small sizes of all equipment and these sizes were not always available. This was supported by quotes from the participants;

*“Babies delivered at home (self-referral baby) weighing 600mg with severe RDS, and needed to be resuscitated…there was no size 0 mask when I needed it…”* (Participant 4 from hospital A).

*“Their feeding tube sizes get finished fast from the hospital’s pharmacy and will be forced to order from other hospitals, which usually take time and these babies needed to be fed.”* (Participant 1 from hospital C).

Perinatal deaths from spontaneous preterm labour are sometimes caused by lack of adequate neonatal facilities and specific equipments in rural areas [4].

### Sub-theme 1.5: A need for constant training of midwives regarding care of preterm babies

All the midwives who are working in neonatal unit should undergo specific training for neonatal, specifically preterm complications management. Midwives who were involved in this study were not trained as neonatal nurses or on LINC which was the course, taught to all midwives working in maternity/neonatal ward. LINC provides health care personnel with knowledge and skills on the diagnosis and management of different conditions of small and sick neonates. Relevant quotation from participant 5 from hospital A and participant 4 from hospital B supports this notion:

“…there are only general midwives, no NICU trained nurses and some of the tasks require skills for intensive care, nursing is improvised and compromised. The institution improve our skills and knowledge by undergoing trainings like ESMOE, HBB, LINC and also conduct in-service education amongst ourselves every Wednesdays. But the shortage of staff, make it difficult for us to attend these trainings.”

All midwives should be constantly trained on how to perform these procedures. The Limpopo Initiative for New-born Care (LINC) training was made available for nurses and doctors to improve their skills, knowledge and confidence to recognise, manage and treat small and sick new-borns; this programme has improved the standard of quality new-born care in Limpopo Province [7].

### Sub-theme 1.6: The importance of proper structure to house preterm babies which will lead to quality care provision

Participants indicated the need for having proper unit structure for caring preterm babies as improper crowded units were leading to potential complications to preterm babies. The noise and light disturbs the normal development of the babies, midwives could not keep the lights off in the neonatal units because it was needed when performing procedures. One hospital was under construction during the period of data collection and midwives indicated that the noise produced from construction also disrupts with the normal development of the preterm babies as they do not need too much noise. The following were quoted with regard to unit structure to house preterm babies:

*“The environment is not conducive for care. It is like a one-roomed house with seven beds one closed incubators and three open incubators. If more than seven babies are admitted, some babies will be kept on cribs and the room will be congested and overcrowded…”* (Participant 3 from hospital A said).

*“There is no side ward to nurse very ill or preterm baby with suspected infectious conditions. When the baby changes condition and requires continuous resuscitation, we do it in front of other mothers; we cannot tell them to go outside because they would still be feeding their babies. There is no privacy…in cases where resuscitation fails, they also see you removing drips and doing other things. This is frustrating for us and as for them they get traumatized by witnessing the death of another preterm baby”* (participant 4 from hospital C).

According to a study conducted in Limpopo Province in 2003 by UNICEF to ascertain the status and availability of new-born care services and infrastructure, it was found that none of the health facilities in the province had level 2 new born care units [7].

## Discussion of Findings

There was higher incidence of perinatal mortality amongst severely preterm babies (61.4%) as compared to moderately preterm babies (22.46%) [5]. Twenty-three midwives who were working in the maternity units of the low resource hospitals shared challenges they faced. They further shared experiences of care provided to preterm infants with the limited resources in the facilities. Midwives displayed knowledge and understanding on what contributory causes of preterm deaths and prevention of complications were. Common complications cited by participants were infections, RDS, hypothermia, hypoglycaemia and jaundice. Participants cited that preterm babies were at high risk of acquiring infection; due to their immature immune system. Midwives ensured that preterm babies were protected from infection through limited contact with other individuals, handwashing and promotion of exclusive breastfeeding [23]. Preterm babies were losing heat very rapidly after birth [12]. The evaporative heat loss in preterm infants during the first week of life is said to be due to the increase in trans-epidermal water losses as a result of their poorly developed epidermis and a relatively large surface area in relation to body weight [24]. Hypothermia has been independently associated with increased energy consumption, neonatal cold injury, poor weight gain and susceptibility to infection that may jeopardise the condition of a neonate [13]. Preserving warmth was accurately maintained by KMC, but when babies need incubators, that was a challenge. In the clinical setting, preterm infants of less than 28 weeks gestation were nursed under a radiant warmer which provides unobstructed access for medical and nursing staff to treat and care for these infants [25]. However, the findings of this study revealed that there was lack of adequate radiant warmers, incubators and KMC rooms were overcrowded. Insufficient KMC rooms is common in most low income countries [26].

Preterm babies were found to be suffering from RDS which is a condition related to prematurity and accounts for 15% morbidity in infants born at 34 weeks and 3.2% in those born at 36 weeks [22]. While most premature babies were born just a few weeks early. These babies could be saved by the skill and competency of the staff, however, midwives who were involved in the provision of care were not trained as neonatal nurses or on Limpopo Initiative New-born Care (LINC). About 50% of preterm babies with a gestational age of 24 to 28 weeks may require intubation and mechanical ventilation to maintain extra-uterine life [13]. This was also supported by Lawn et al., that extreme premature babies, require additional skills, equipments and commodities that are critical, ranging from bag and mask, controlled intravenous fluid-giving sets, to nCPAP and surfactant [27]. For babies with RDS, safe monitoring of oxygen saturation was required to be monitored with pulse oxymeter However, these equipments were often unavailable. The equipments necessary to care for preterm babies are incubators, wall suction units, transcutaneous bilirubin meter, ventilators, nasal nCPAP, head boxes, pulse oxymeters, apnoea monitors, oxygen blenders, intravenous infusion controller, resuscitation equipments and feeding equipments [23]. Findings of this study were similar to the study by Thommesen who found midwives’ challenges in providing quality maternal and neonatal care as poor working conditions, feeling of insecurity and frustration at work [15].

It was clear from findings that preterm babies needed individualised care, by competent human resources. When providing care to preterm babies the individualised care was preferred, however, the structure of the unit, shortage of staff and equipments was a barrier. McNamara indicated that there are various challenges to the management of preterm labour which may require individualised approaches for different patients, using expert committees or guidelines as the backbone of the management plan [22]. In this study, it was difficult to achieve individualised care because of shortage of competent staff, limited equipments, and the required sizes of the equipments and overcrowded units. Reduction of length of hospital stay can be achieved by minimising hospital acquired infections which usually occurs during close contact between patients, preterm babies in this case and accidentally using the same equipment in infants. The use of semi-private rooms for neonates who are critically ill for individualized [16].

A study conducted in the United Kingdom indicated that across nine countries in sub-Saharan Africa and South East Asia, in each country setting and for each cadre of health care provider, there was a significant improvement in knowledge and skills after receiving a short competency-based training package in emergency obstetrics and early newborn care [28]. The findings by show that there is a need for continuous training of midwives in newborn care [28]. In South Africa, Emergency Obstetric and Neonatal Care (EmONC) was adopted to include a substantial module on intubation as part of the resuscitation module; the package is known as Essential Steps in Managing Obstetric Emergencies (ESMOE), and it is compulsory for all maternity staff in improving knowledge and skills [29]. Again, the Limpopo Department of Health, was providing the course on Limpopo Initiative Neonatal Care to all those working in maternity and neonatal units. The LINC provides health care personnel with knowledge and skills on the diagnosis and management of different conditions of small and sick neonates. However, midwives who participated in this study were not trained as neonatal nurses, paediatric nursing or on Limpopo Initiative New-born Care (LINC). Researchers noted that to provide capacity for staff, continuous in-service education were scheduled but attendance was poor due to staff shortage especially when the unit was busy.

## Conclusion

The findings of the study revealed that midwives who are working at resource limited health facilities were encountering challenges when providing care to preterm babies. This was due to staff shortage and lack of necessary and appropriate equipments when caring for preterm babies, Due to inadequate equipments, the standard of care was compromised. Preterm babies developed complications which needed specialised care by skilled and knowledgeable midwives. It was recommended that to save the lives of the preterm babies it is of great importance to improve the skills and competence of midwives through training on LINC, HBB and NICU. The health facilities need to plan and send at least one midwives for LINC training every year and one nurse for NICU every two years. Operational managers should be involved in the ordering and supply of necessary equipment and the correct sizes to use on preterm babies. In-service trainings should be conducted weekly within the facility to equip nurses with updated knowledge and skills on management of different conditions and the use of available machines. Mothers should be taught on measures to prevent infection in the neonatal unit such hand washing, using of surgical spirit for cord care; and also to teach them about the importance of practicing KMC (Kangaroo Mother Care). Future research should be conducted on the same topic in different settings to generate more knowledge on the phenomenon.

## Additional File

The additional file for this article can be found as follows:

10.5334/aogh.2555.s1 Transcript. Interview with the Fifth Participant, Midwife (hospital A).

## References

[1] United Nations Children’s Fund South Africa (UNICEF SA). Improving Newborn Care in South Africa, Lessons learned from Limpopo Initiative for Newborn Care (LINC). Pretoria: UNICEF South Africa; 2011.

[2] Save The Children. Surviving the first day. State of the world’s mothers; 2013.

[3] Pattinson RC, Rhoda N. Saving babies 2012–2013. Ninth report on perinatal care in South Africa. Pretoria: Tshepesa press; 2014.

[4] Pattinson R. Challenges in saving babies-avoidable factors, missed opportunities and substandard care in perinatal deaths in South Africa. South African Medical Journal. 2003; 93(6): 450–455. 12916386

[5] Goswami S, Sahai M. Premature birth: An Enigma for the Society? European Journal of Medicine. 2014; 6(4): 215–225. DOI: 10.13187/ejm.2014.6.215

[6] United Nations Children’s Fund South Africa (UNICEF SA). Improving Newborn Care in South Africa, Lessons learned from Limpopo Initiative for Newborn Care (LINC). Pretoria: UNICEF South Africa; 2011.

[7] Chopra M, Daviaud E, Pattison R. Saving lives of South Africa’s mothers, babies and children: Can the health system deliver? Lancet. 2009; 29–39. DOI: 10.1016/S0140-6736(09)61123-5 19709729

[8] Pattinson RC (ed). Saving Babies 2006–2007: Sixth report on perinatal care in South Africa. Pretoria: Tshepesa Press; 2009.

[9] World Health Organization. Born Too Soon. The Global Action Report on Preterm Birth. North America: WHO Press; 2012.

[10] Liu L, Johnson HL, Cousens S, et al. Global, regional, and national causes of child mortality: an updated systematic analysis for 2010 with time trends since 2000. The Lancet. 2012; 379(9832): 2151–2161. DOI: 10.1016/S0140-6736(12)60560-1 22579125

[11] Wisanskoonwong P. Midwifery primary health care groups during childbearing epublications@scu, Southern Cross University; 2012.

[12] Fraser D, Cooper MA, Nolte AGW, Myles MF. Myles textbook for midwives. Churchhill Livingstone: Elsievier Health Sciences; 2010.

[13] Joseph RA. Prolonged mechanical ventilation: challenges to nurses and outcome in extremely preterm babies. Critical Care Nurse. 2015; 35(4): 58–66. DOI: 10.4037/ccn2015396 26232802

[14] Brophy DM. Occupational challenges faced by nursing personnel at a state hospital in Cape Town. South Africa: Cape Peninsula University of Technology; 2015.

[15] Thommesen T. Midwives’ challenges in providing quality maternal and new-born care in the health centres in Addis Ababa, Ethiopia, Centre for International Health. Norway: University of Bergen; 2014.

[16] Floyd AMD. Challenging designs of neonatal intensive care units. Critical Care Nurse. 2005; 25(5): 59–66. 16183753

[17] Ntuli ST. Evaluation of maternal mortality Limpopo initiatives Research day presentation. Department of Public Health; 11 2012.

[18] Patel RM, Kandefer S, Walsh MC, et al. Causes and timing of death in extremely premature infants from 2000 through 2011. New England Journal of Medicine. 2015; 372(4): 331–340. DOI: 10.1056/NEJMoa1403489 25607427 PMC4349362

[19] de Vos AS, Strydom H, Fouchè CB, Delport CSL. Research at Grass Roots for the Social Sciences and Human Service Professions. Pretoria: Van Schaik Publishers; 2011.

[20] Creswell JW. Research Design: Qualitative, quantitative, and mixed methods approaches. Dorset Press, Henry Ling Limited; 2016.

[21] Lincoln YS, Guba EG. Naturalistic inquiry. Beverly Hills: Sage; 1985 DOI: 10.1016/0147-1767(85)90062-8

[22] McNamara HM. Problems and challenges in the management of preterm labour. BJOG: An International Journal of Obstetrics and Gynaecology. 2003; 110(s20): 79–85. DOI: 10.1046/j.1471-0528.2003.00042.x 12763118

[23] Essential Newborn Care. Recommended standards for essential neonatal care. 2012 http://studylib.net/doc/6874711/recommended-standards-for-essential-neonatal-care.

[24] Kong YS, Medhurst A, Cheong JLY, Kotsanas D, Jolley D. The effect of incubator humidity on the body temperature of infants born at 28 weeks’ gestation or less: a randomised controlled trial. Neonatal, Paediatric & Child Health Nursing. 2011; 14(2): 14.

[25] Bradshaw D, Chopra M, Kerber K. Every death counts: Saving the lives of mothers, babies and children in South Africa. Every Death Counts. 2008; 1–15. 10.1016/S0140-6736(08)60564-4 18406864

[26] Gondwe A, Munthali A, Ashorn P, Ashorn U. Investigating Preterm Care at the Facility Level: Stakeholder Qualitative Study in Central and Southern Malawi. Maternal and Child Health Journal, 2016; 20(7): 1441–1447. DOI: 10.1007/s10995-016-1942-z 26976282

[27] Lawn JE, Gravett MG, Nunes TM, Rubens CE, Stanton C. Global report on preterm birth and stillbirth (1 of 7): definitions, description of the burden and opportunities to improve data. BMC Pregnancy and Childbirth, 2010; 10(1): S1 DOI: 10.1186/1471-2393-10-S1-S1 20233382 PMC2841772

[28] Ameh CA, van den Broek N. Making it happen: Training health-care providers in emergency obstetric and newborn care. Best Practice & Research Clinical Obstetrics & Gynaecology. 2015; 29(8): 1077–1091. DOI: 10.1016/j.bpobgyn.2015.03.019 25911056

[29] Ameh CA, Kerr R, Madaj B, et al. Knowledge and skills of health care providers in sub-Saharan Africa and Asia before and after competency-based training in emergency obstetric and early newborn care. PloS One. 2016; 11(12): e0167270 DOI: 10.1371/journal.pone.0167270 28005984 PMC5179026

