# Supplementary material for: Provision of Care to Preterm Infants at Resource Limited Health Facilities of Mopani District, South Africa
Source: Ann Glob Health. 2020 Feb 3;86(1):10. doi: 10.5334/aogh.2555 (PMC7006596; doi:10.5334/aogh.2555)
Supplement: Transcript. — Interview with the Fifth Participant, Midwife (hospital A). [file agh-86-1-2555-s1.pdf]

TRANSCRIPT: INTERVIEW WITH THE FIFTH PARTICIPANT, MIDWIFE (HOSPITAL A)

The researcher introduced herself to the participant; showed the participant a permission letter granted by the hospital; provided the participant with an information sheet which included the purpose of the study, objectives, and the ethical consideration. The researcher allowed the participant time to go through the information sheet and also explained the content of the information to make sure that the participant understood everything before signing the consent form. The researcher also explained to the midwife that no names should be mentioned during recordings as this may violate the right of anonymity.

Researcher: "Sister, what challenges do you face when you are nursing premature babies?"

Midwife Lth A: "Due to their systems which are not well developed, premature babies suffers from hypothermia which can result into more serious conditions like hypoglycaemia and respiratory distress. Preterm babies should be nursed in servo-controlled incubators. The available incubators are not well functional, they need to be checked all the time because you can set the required temperature and they change and becomes too cold or too hot."

Researcher: "So the prematurity of these babies' organs lead to them having different clinical problems like respiratory distress and hypoglycaemia as you have mentioned. What other clinical problems do these babies experience that makes caring for them challenging to you?"

Midwife Lth A: "They also have a problem of feeding intolerance, you may find that they are failing to finish the prescribed feeds for the day, or they finish feeds but vomits every now and then, this affects their weight gain, if not feeding well the baby will not gain weight instead they will lose or weight remains the same."

Midwife Lth A: "Long hospital stay in preterm babies is a common challenge."

Researcher: "What causes long stay? How does it become a challenge? Can you explain in detail?"

Midwife Lth A: "when the babies are admitted they are seen by multidisciplinary team including nurses, doctors, dieticians, physiotherapists and mothers...Long stay can be due to being handled by multiple personnel; in this case they develop infection."

Midwife Lth A: "For example: a preterm baby admitted at 0.9kg or 0.88kg should stay in the hospital and grow until they reach the weight of 1.8kg. Stay becomes very long because within their first ten days of life, all babies start to lose 10% of the birth weight. Sometimes the baby vomits, may have infection and should be on drip and be given antibiotics...Problems like vomiting and infection cause poor weight

gain... When you nurse the neonate, you should nurse the mother as well. Mothers get bored by staying in the hospital, sometimes the family and friends don't visit them, and you are supposed to act as a midwife and a relative."

Midwife Lth A: "They need to be measured head circumference every week for early detection of hydrocephalus. Weight monitoring is done daily to see if the baby is gaining adequately, if not gaining and feeding well, bloods investigations need to be done to identify the cause. Weight gain is sometimes affected when you start feeds and the baby start having abdominal distension and feeds need to be omitted. Mothers get stressed when their babies are not gaining weight. Due to prematurity they have slow digestion and can go 3-4 days without passing stools, it is also stressing on the mother."

Midwife Lth A: "Preterm babies are fed three hourly. Those who cannot feed well by cup are fed using feeding tubes. Sometimes a baby can be fed at 09h00 via feeding tube, and when the mother comes to give 12h00 feeds she may find that the baby has removed the tube, or the tube came out during vomiting. As a midwife, you will have to re-insert another tube, which is irritating to the mother because mothers think that inserting tubes hurts their babies."

Researcher: "What are the standard procedures do you practice as midwives to promote care for preterm babies?"

Midwife Lth A: "The multidisciplinary team is taught on practicing antiseptic hand washing before and after handling each baby."

Researcher: "Okay you teach everyone to wash their hands every time the handle the babies. Do you encounter any challenge with regard to practicing hand washing?"

Midwife Lth A: "... they sometimes forget to wash hands, they can handle baby A then move to baby B and so on without washing or disinfecting hands. This increases the risk of cross infection amongst the babies. Mothers may come in the unit and find that the baby has long been crying they concentrate more on the baby's cry and go straight to where the baby is lying, forgetting to wash hands...D-germ (surgical spirit) is put on each bed for spraying on hands before and after handling the baby for infection control. It is also put at the ward entrance so that everyone who comes in disinfects hands. Mothers are advised to wash hands, then change nappy, take it to the bin, come back and repeat the same procedure of washing hands."

Researcher: "What happens when they forget to wash hands? What effects does it have to the babies?"

Midwife Lth A: "Like I have already said before, the babies will stay longer in the hospital because they will develop infection...Necrotising enterocolitis is a common infection in preterm babies, caused by not washing hands and prolong hospital stay, this condition causes poor weight gain in neonates...and prolonged stay increases the workload for us as the midwives caring for these babies"

Researcher: "Are there other challenges that perhaps are the results of long stay? I mean you have mentioned

the issue of mothers getting tired of staying in the hospital and wanting to go home, and you also added to say it increases the work load for you."

Midwife Lth A: "Prolonged hospital stay also results in over expenditure because more treatment will be given, more equipment will be used in one baby...Over expenditure is a critical problem. If one baby removes feeding tube every now and then it means more tubes are being used for one baby, this is over spending according to the hospital's budget. Normally feeding tubes must be changed after 6 hours. The size of tubes used in preterm babies are short, they can be finished in dispensary and get to be ordered from other hospitals which is a process that takes long."

Researcher: "Please tell me about the administrative challenges that you face."

Midwife Lth A: "These babies are still developing, they don't need disturbances."

Researcher: "What do you mean when you say they don't need disturbance?

Midwife Lth A: "They don't need noise. They don't need bright light...The hospital is under construction and it produces too much noise which interrupts with normal development. Light disturbs them as well; we cannot keep the lights off because it is needed when working and performing procedures like collection of blood, insertion of drips, etc. The development is also disturbed when we wake them up during feeding hours or when the health care workers want to check them...People can look at them as just tiny babies but they need to rest."

Researcher: "Okay I hear you are saying they do not need to be disturbed by either light or noise, which are the things you cannot avoid because your hospital is under construction and light is needed for routine work. What other challenges do you have in relation to working environment?"

Midwife Lth A: "The incubators are available. We are using servo-controlled ones. The problem we have with them is that they are not well functional...you can set the temperature to be average, and it will either become too hot or cold, affecting the condition of the baby. If you don't check them, you may find the baby being hypothermic...hypothermia causes deaths in preterm babies because they are not well matured...they are not well developed."

Researcher: "When we were talking about long stay we spoke about mothers longing to go home...what else can you tell me about mothers of these preterm babies? You said when you nurse the baby; you also nurse the mother as well? How do you do that?"

Midwife Lth A: "Mothers of the preterm babies need counselling from the day of admission. They becomes traumatised due to delivering a very tiny baby, they become stressed and even develop depression...they need to be cared for as well...its challenging, sometime they fail to co-operate...Sometime a mother can see another baby with the same weight as her baby's being transferred to kangaroo mother care (KMC) unit and hers still having drips, feeding tubes, etc., and start worrying and end sometimes end up being psychotic."

Midwife Lth A: "... This is why the mother should be involved in every act for them to understand every management to be executed. Psychological stress in mothers affects breast milk production. Everyone nursing preterm babies should be patient and understand that care should involve the mother. As midwives we should emphasise hand washing. Mothers should hold the babies for at least 30mintues after feeding to avoid aspiration and vomiting."

Researcher: "How do you cope with the work load that increases with prolonged stay?"

Midwife Lth A: "We try our best but it is hard ...The hospital is experiencing a problem of staff shortage...we do lot of work...we get exhausted...and another problem is rotation of staff which is also a challenge because some midwives who come to the nursery are not LINC (Limpopo Initiative Newborn Care) trained and some have no love for babies...staff rotation lead to allocation of midwives who do not have interest in neonates and it is a challenge to work in areas you are not passionate of...let alone working with someone who is not interested in what they are doing."

Researcher: "You are saying with staffing you are experiencing a challenge of shortage and having to work with midwives not interested in neonates, what other problems have you encountered while working with preterm neonates, something we didn't talk about?

Midwife Lth A: "... Uhmm...we have many challenges, most are related to the conditions of the babies that are caused by immaturity of their body organs...conditions such as respiratory distress, babies changes without giving you a sign... uhm yes it is not easy working with these neonates ..."

Researcher: "... Okay I have understood your challenges and that will be all for now, thank you very much for your cooperation."
